# Characterization of new cellulose fiber extracted from second generation Bitter Albizia tree

**DOI:** 10.1038/s41598-024-51719-y

**Published:** 2024-01-19

**Authors:** T. P. Sathishkumar, Mohd Asif Shah, Hitesh Panchal, Kamal Sharma, R. Gopinath, M. R. Sanjay, Suchart Siengchin, L. Rajesh Kumar, G. S. Rampradheep

**Affiliations:** 1 Department of Mechanical Engineering, Kongu Engineering College, Erode, Tamilnadu India; 2 Bakhtar University, Kabul, Afghanistan; 3 Centre of Research Impact and Outcome, Chitkara University Institute of Engineering and Technology, Chitkara University, Rajpura, 140401 Punjab India; 4 Division of Research and Development, Lovely Professional University, Phagwara, Punjab 144001, India; 5 Department of Mechanical Engineering, Government Engineering College patan, Katpur, Gujarat India; 6 Department of Mechanical Engineering, GLA University, Mathura, India; 7 Department of Civil Engineering, University College of Engineering, Tindivanam, Tamil Nadu India; 8 Natural Composite Research Group Lab, Department of Materials and Production Engineering, The Sirindhorn International Thai-German Graduate School of Engineering (TGGS), King Mongkut’s University of Technology North Bangkok, Bangkok, Thailand; 9 Department of Mechanical Engineering, KPR Institute of Engineering and Technology, Coimbatore, Tamilnadu India; 10 Department of Civil Engineering, Kongu Engineering College, Erode, Tamilnadu India

**Keywords:** Environmental sciences, Materials science

## Abstract

The present work examines the physical, thermal tensile, and chemical properties of wood skin fibers obtained from second generation Bitter Albizia (BA) tree skin. Chemical characterization of BA fibers showed the presence of various chemical contents such as cellulose of 74.89 wt. %, hemicellulose of 14.50 wt. %, wax of 0.31 wt. %, lignin of 12.8 wt. %, moisture of 11.71 wt. %, and ash of 19.29 wt. %. The density of BA fibers (BAFs) was showed 1285 kg/m^3^. XRD analysis of BAFs showed a crystallinity index (CI) of 57.20% and size of crystallite of 1.68 nm. Tensile strength and strain to failure of BAFs examined through tensile test were 513–1226 MPa and 0.8–1.37% respectively. TGA portrayed the thermal steadiness of BAFs as 339 °C with 55.295 kJ/mol kinetic activation energy, its residual mass was 23.35% at 548 °C. BAFs with high CI, less wax content, and better tensile strength make more suitable for making polymer matrix composites. SEM images of the BAFs surface depicted that the fiber outer surface has more rough which shows that they can contribute to hige fiber-matrix adhesion during composites preparation.

## Introduction

Due to the increase in adverse chemical reactions in the environment, research activities focus to swap synthetic fibers and its composites with natural or bio-based fibers and its composite. Synthetic fibers such as carbon, glass. nylon and kevlar have been widely used for making polymer composites for more than three decades. Increasing the demand of natural biodegradable fiber in the industries and the global market, it should need to find new natural fiber^1,2^. Main purpose of this study investigates the mechanical, chemical, thermal and properties of novel natural fibers pull out from the skin of BA trees. Albizia is a tree of fabaceae family. It grows prominently in South and East Africa, Sudan and Ethiopia. It is also found in India and other countries like Sri Lanka, Bangladesh, etc. Performing the required analysis on integrated properties ensures the suitability of various natural fibers and it is very significant to have basic information for making composites. Therefore, studying the characteristics of such fibers through research plays an important role in introducing new natural fibers to the engineering and technology field. A standardized testing process for performing structural analysis of natural fibers was followed by many researchers^3^. The advantage of natural fibers is high compared with synthetic fibers like carbon, glass and kevlar, including lower production costs, reduced environmental impact, lighter weight, greater accessibility, and resistance to corrosion^4^.

Many engineering industries, including construction, automotive, aerospace, electrical, marine, and home utilities the natural fiber reinforced polymer composites in a broader extent^5,6^. Natural fibers’ mechanical qualities were determined solely by their chemical make-up, growth region, source, age, and extraction technique^7,8^. Many researches that the microfibrils are oriented spirally in the fiber axis, it exhibits rigid behaviour. The efficiency of reinforcing the natural fibers increase with increase in the length-to-diameter ratio (i.e. aspect ratio) of natural fiber over the critical ratio at which applied load or stress transfer between matrix and natural fiber occurs completely before the failure of composite samples. However, higher aspect ratio leads more fiber entanglement during the composite manufacturing processing and occurred poor dispersion^9^. In this way, many researchers have extracted new biofibers such as Pandanus Tectorius^8^, Acacia leucophloea^10^, Sansevieria Ehrenberg^11^, Prosopis juliflora^12^ and *Agave gigantea*^13^, using qualitative procedure. FTIR spectroscopy, XRD, TGA^10^ and Single fiber testing was adopted to characterize the fibers. These fibers are used to develop the fiber reinforced polymer matrix composites. In many cases, the natural plant fibers like cotton, sisal, hemp, and jute are also used as reinforcements to produce a hybrid composite^1,2^.

Plants that create such natural fibers, on the other hand grows exclusively in certain places based on natural circumstances. It is important to test new fiber with advisable properties that may guarantee the development of green composites. It is expected that the future requirements for unique natural fibers will expand as new products development and technologies. The requirement and demand of natural fiber are continuously increased in the world market. Compensating the demand of natural fiber in the work marked, the new plant fiber should be identified and characterized before being used in composite developments. A new plant fiber is identified from a plant of *Bitter Albizia tress.* The current study investigates the physical, thermal, tensile, and chemical properties of unique BA bark fiber, which has not been reported in the literature. BA fibers extracted manually from plant stalks and are subjected to mechanical property testing like tensile behaviour. Alongside, TGA, DSC, XRD, and FTIR, and morphology of fiber surface using Scanning Electron Microscopy are investigated in the present study. The BA tree was collected from agricultural land as per the Tamilnadu agricultural institution guidelines, Tamilnadu, India.

## Materials and methods

### Bitter Albizia plant and its fiber extraction

**Figure 1:**
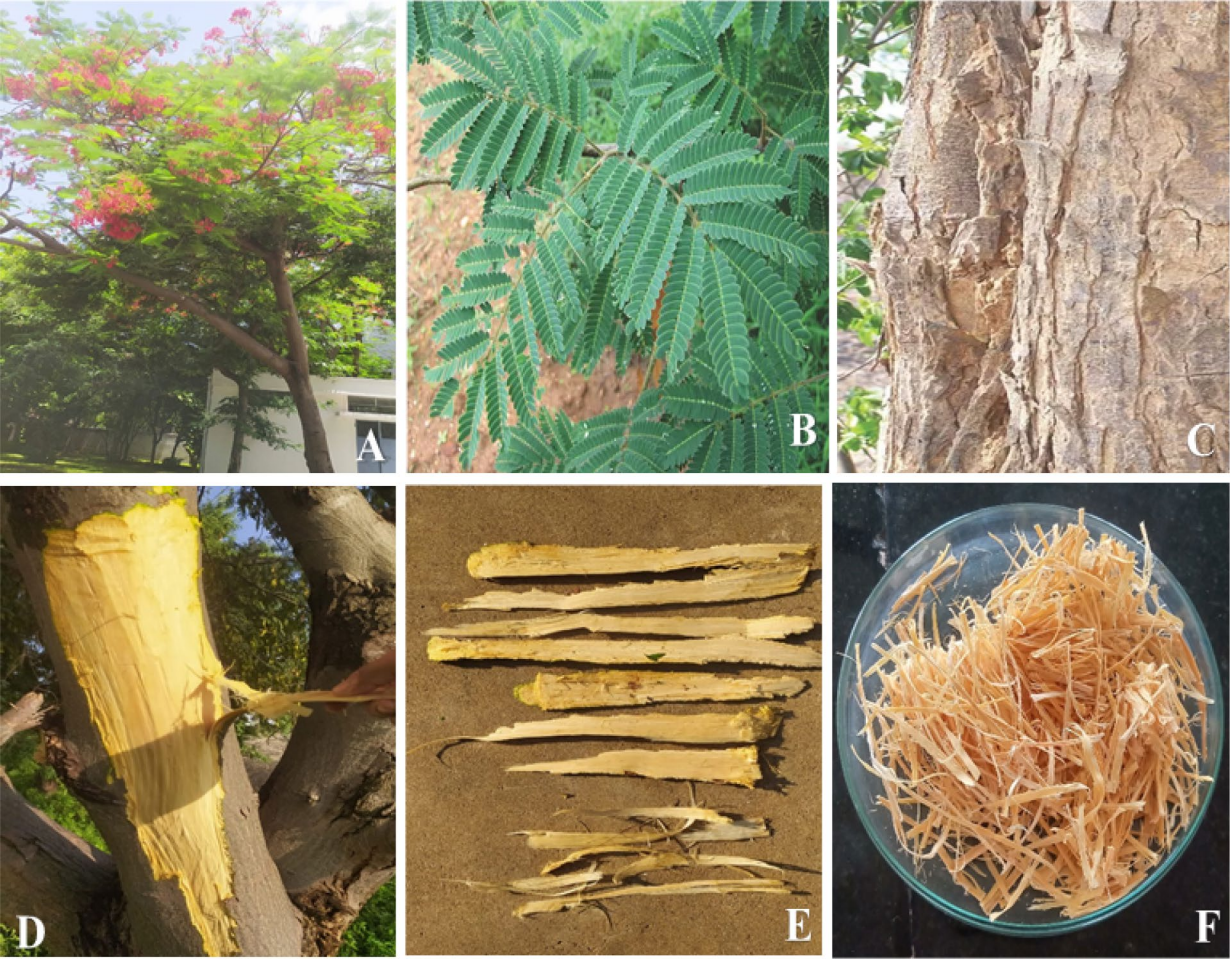
(**A**) Bitter Albizia (BA) Tree, (**B**) Leaf Twig of BA, (**C**) Bark in BA tree (hard skin), (**D**) Peeling of BA skin, (**E**) BA skins and (**F**) BA skin fibers (BAFs).

*Bitter Albiza* tree (Fig. 1A) belongs to Fabaceae family. It is abundantly available in South and East Africa. The Bitter Albizia tree was cultivated and harvested as per the guideline stated in Institute of Forest Genetics and Tree Breeding by Indian Council of Forestry Research and Education, Coimbatore, Tamilnadu, India. The trees are collected from an agricultural land in Perundurai, Erode district, Tamilnadu, India. The GPS locations of the collected BA tree skin are in East of 77° 35′ 26.1492″, North of 11° 16′ 5.0664″, Latitude of 11.268074 and longitude of 77.590597. The prior permission was approved by concern authority for collecting this tree. Its leaves are in small size ranging between 2 and 8 mm (Fig. 1B). Figure 1C shows the hard outer skin of BA tree and it peeled off manually (Fig. 1D) for fiber extraction. Figure 1E shows the collected skin fiber extraction. Figure 1F shows the extracted fibers for characterization. Restoration processes are adopted to remove plant fibers (Table 1). Bitter Albizia (BA) plant bark is waterlogged in stored water for three weeks to discrete the fibers using water soaking process^11^. The extracted skins fiber is dried at room temperature for three more days and prepared to use for the future study.

**Table 1:** Natural fibers extraction methods.

Name of the fiber’s	Method of extraction	Refs.
Acacia leucophloea Bark fiber	Water with microbial degradation	^10^
Hibiscus vitifolius fiber	Water retting	^14^
Furcraea Foetida fiber	Water	^15^
Cyperus Pangorei fiber	tableWater	^16^
Carica papaya fiber	Water	^17^
Hemp fiber	Hydrothermal retting method	^18^
Flax fiber	Enzymatic method	^19^
Jute or Urtica Dioica fiber	Dew/microbial retting	^20^
Acacia Planifrons fiber	Water/ultrafine long metal teeth	^21^
Ficus racemosa fiber	Water	^22^
Banana fiber	Steam explosion mehtod	^23^
Sisal fiber	Mechanical decorticator	^24^
Elephant grass fiber	Chemical soaking	^25^
Cotton fiber	Alcohol chenical extraction method	^26^

### Chemical analysis of BAFs

Standard test procedures were adopted to calculate the various chemical components or properties of BAFs, including wax, hemi-cellulose, pectin, cellulose moisture, and ash content^7^. BAF density is obtained using the meltbertoledoxsz05 balances method of (Machine maker and mode: Mettler Toledo and xsz05) testing. The moisture level of BAF was examined using the electrical humidity analysis, and the ash component was quantified according to ASTM E1755-01 standard using Cubis® II (Sartorious, model MA45). The wax mixture is calculated in the Conrad method. Pectin is analyzed in the form of ammonium oxalate KOH(Potassium hydroxide) amalgamation method.

### Moisture

Moisture analysis is performed to see the humidity in the inner and outer fiber surface which can affects the mechanical behaviour of natural plant fibers, particularly in the fiber-matrix connection. The test was performed by measuring approximately 5 g of sample in a towel-filled petrol container. It is also stored in a dry place in an air oven of 100 ± 20 °C. Then, the samples were cooled in automatic desiccator for period of 25 min and their weight of the sample was calculated. Again, the BA fibers were dried for 30 min, cooled in a autmomatic desiccator for 25 min and again their weight was calculated. This process was continuously repeated until a saturated value of weight was obtained during two consecutive trials. The moisture lavel in the fiber is calculated by using Eq. (1).

1 $${\text{Moisture}}\,\,(\% \,\,{\text{by}}\,\,{\text{weight}})\, = \,\frac{{{\text{W}}_{1} - {\text{W}}_{2} }}{{{\text{W}}_{1} - {\text{W}}}} \times 100\,,$$

where W is dish weight in grams, W_1_ is dish weight in grams + weight before dying in grams, W_2_ is dish weight + weight after dying in grams.

### Ash

Ash test is done to calculate the total content of the ash filled inside the BA fiber. The test was performed using a 5 g of sample in a towel container. The inside temperature of the container was maintained as 550 °C. Heating rate in the oven was maintained as 10 °C/min, so that the formed ashes are carbon-free. Weight of the fibers were then recorded using standard measuring practices. The Ash content is calculated by Eq. (2):

2 $${\text{Total}}\,{\text{ash}}\,\,{ = }\,\frac{{{\text{(W}}_{2} - {\text{W}}_{1} ) \times 100 \times 100}}{{{\text{(W}}_{{1}} - {\text{W)(100}} - {\text{m)}}}}\,,$$

W_1_ = crucible + sample weight (g), W_2_ = crucible weight + ash content (g), W = the empty crucible weight (g), m = moisture % of the sample.

### Pectin

A beaker of 1000 ml capacity have mixed 50 g of BAFs and 400 ml of 0.005 N HCl. The beaker was heated upto 90 °C for 2 h and boiled with water of 400 ml. This was cooled and transfered to a volumetric flask of 500 ml. Here, add 200 ml of distiller water and 10 ml of NaOH in the beaker, it allows overnight. Finally add acetic acid and calcium chloride of 50 ml and 25 ml in the beaker, it is boiled for 3 min and filtred. The filtered residual was used to calculate the pectin content of BAFs.

### Cellulose

Cellulose is a structural polysaccharide present in plants. Initially the samples were appropriately scissored. They were then freezed by keeping in a liquid nitrogen bath and converted into fine powder. This samples were weighed approximately ranging between 0.5 and 1.5 g of samples and they were transferred to tubes of 50 ml capacity. Then the BAF samples were heated in a hot water at 100 ºC for 100 min before taking them out. Following the heating, a 10 ml of hydrogen peroxide solution (5%) was added into the tube. Again, the BA fibers were placed in hot water bath at 75 °C for 40 min and the samples were cooled in the room temperature for 60 minuts. The samples were then packed with filter paper and they were dried in an incubator at 65 °C temperature until reaching the constant weight. The weight of the dried insoluable samples were weighed which showed the present of cellulose content and preserved in the centrifugal tubes.

#### Hemi-cellulose

They are a combination of plant polysaccharides and lower molecular weight than cellulose and soluble in diluted alkali. They are employed in the making paper and ethanol. In an acetate buffer solution, the pulp was dipped in a dry solid to liquid ratio content of 1/60 (g/ml). Following the process, the holocellulose substance was rinsed and filtered with distilled water, and followed to dried in an hot air oven for 20 h at 40 °C. Sodium sulphite and Neutral detergent solution were used wet the cellulose for a period of three days. Three volumes of ethanol were used to precipitate hemicellulose, which was then filtered, freezeed and dried.

### Lignin

Lignin is an important organic polymer found in the walls of various types of cells. It includes biological tasks such as mechanical support, water transport, and stress resistance. For the lignin test, a variety of chemicals were utilised, including acid, peroxide, caustic soda, and water. A small amount of pulp was removed and placed in a conical flask. The 85% of organic acid (formic and acetic acid mixture of 70 and 30 by volume) was added to the flask at a fiber to liquid ratio of 1:8 and left to boil for two hours on a hot plate. The flask and its substances were cooled to room temperature after 2 h. The fibers were filtered through a Buckner funnel and then washed in 80% acid before being rinsed in hot H_2_O. The delignified fibers were then bleached for two hours in an extremely precarious bath at 8 °C using a 14 ml of 35% H_2_O_2_ solution. Following the pulping and delignification process, the wasted liquor was heated to 105 °C. The H_2_O was used to precipitate lignin that had been dissolved in acid.

### Physical analysis of BAF’s—density

A single long fiber was sliced into short length and kept on a microscope sliding table. It was projected to a screen using a microscope. The dimensions were measured in millimetre using a ruler. By measuring the weights and dimensions of the object, the linear density was computed. A total of 100 fibers were examined in order to estimate the average BA fiber diameter and 10 fibers were tested in order to calcalted the average linear density. Using Eq. (3), the volume density of the natural fiber was calculated using the average linear density and average diameter of fibers^27^.

3 $${\rho }_{f=}\frac{M}{\frac{\pi {d}^{2}}{4}l},$$

where M = mass of a natural BA fiber (grams), d = average diameter of the BA fiber (m), and *l* = BA fiber length (m).

### FESEM

The surface morphology of BAFs was analyzed through *FESEM* (Field emission scanning electron icroscopy*)* with high resolution (Model: Thermo Scientific Apreo S). The BA fiber surface was coated with gold of thin layer before imaging. The gun voltage of 30 kV was used to capture the image of the BAFs surface.

### FTIR

The Shimadzu spectrometer was used to record the FTIR (Fourier transform infrared spectroscopy) spectrum of BAF’s. The BAF samples were milled using a ball milling machine until they were reduced to a powder. This BA fiber powder and bromide of potassium crystals was mixed thoroughly and then the mixture was compacted into a pellet. FTIR data were collected using the attenuated total reflectance mode, and the scan rate was kept at 32 scans per minute while the resolution remained at 2 cm^–1^^14^ over the frequency range of 4100 to 500 cm^–1^.

### XRD

The X-ray diffraction (XRD) method has been commonly used to know the composition and structure of crystalline materials. The XRD of BAFs was explored (powder XRD) with help of X’Pert-Pro diffractometer^1^.

4 $${CI}_{1/4}= \frac{{H}_{002}- {H}_{am}}{{H}_{002}},$$

where *H*_21.72_ and *H*_15.81_ are the higher peaks at 2θ = 21.72° and 15.81°, respectively. CS (i.e. Crystallite size) is calculated using the deflection pattern of the cellulose’s 200 lattice planes. Using Scherrer’s formula, the CS was computed as follows in Eq. (5).

5 $${CS}_{1/4}=\frac{K\lambda }{\mathrm{B*cos}\theta },$$

where *K* is Scherrer’s constant (0.89), *β* = half width of the radian, *λ(delta)* = the wavelength of radiation (0.15406 Å), and *θ (Theta)* = Bragg angle in radian.

### TGA (thermogravimetric analysis) and DTG (derivative thermogravimetry)

TGA/DTG are done to find the substance weight and its rate of change of phase in term of curve was recored when the BA fiber sample is heated in a controlled nitrogen atmosphere contition. The various stage of BAF degradation were calculated using Jupiter thermogravimetric analysis spectrometer (Makea and Model: STA449F3-NETZSCH, Country: Germany). This device contains a heating furnace and sample of BA fibers placed on a idle crucible attached to an accurate balance. The sample of BA fiber was heated between 36 °C and 650 °C by 10 °C/min heating rate^14^. To prevent the effects of oxidation or burning, the fiber sample was burnt in a nitrogen gas environment with 20 ml/min gas flow rate delivered to the furnace. The activation capabilities of BAFs were tested utilising the Broido firm expression (Eq. 6) to determine their thermal stability.

6 $$In\left[In\left(\frac{1}{y}\right)\right]= -\left(\frac{{E}_{a}}{R}\right)\left[\left(\frac{1}{T}\right)+k\right],$$

where R and T are Universal gas constnat of 8.32 J/mol K and Temperature in Kelvin, , *k* and y are constant and the proportion of initial molecules which is not decomposed (w_t_/w_o_), where w_t_ = sample weight at any time t and w_o_ = Fiber sample’s beginning weight.

### DTA (differential thermal analysis)

The BA fiber sample is heated at a controlled place with an inert reference material in the DTA testing. The reference material and sample temperature was risesed by heating. If the sample undergoes a phase transition, the power is absorbed or extracted, and differerence in referenced and sample temperature (T) is determined. A DTA curve shows the temperature difference as afunction of time.

### Single fiber test

The tensile characteristics of the BAFs were tested by cutting them into 100 lengths. The fiber gauge length is 50 mm and a total of 20 samples of single fibers were evaluated. Individual fibers were gripped using a pneumatic gripper during testing, with the testing cross head speed of 0.1 mm/min^11,15^. The force vs. elongation curve of fiber at various gauge lengths was measured using a 1.3 kN load cell, and the data were plotted. The average tensile strain, Youngs modulus, tensile strength, and elongation values were calculated. The tensile sudy was conducted in a laboratory with the room temperature at 25 °C, and the relative humidity (RH) was set at 72%. Finally, the recorded data were analysed to determine the fiber's tensile characteristics. Equation (7) is used to calculate the total cross head travel (displacement (δt)) during fiber testing:

7 $$\frac{\delta t}{F}=\left[\frac{1}{EA}\right]L+C,$$

where F = applied load (Newton), L = gage length (mm), E = Young’s modulus, A = Fiber Cross sectional area, and c = machine compliance.

## Results and discussion

### SEM analysis of BAF

**Figure 2:**
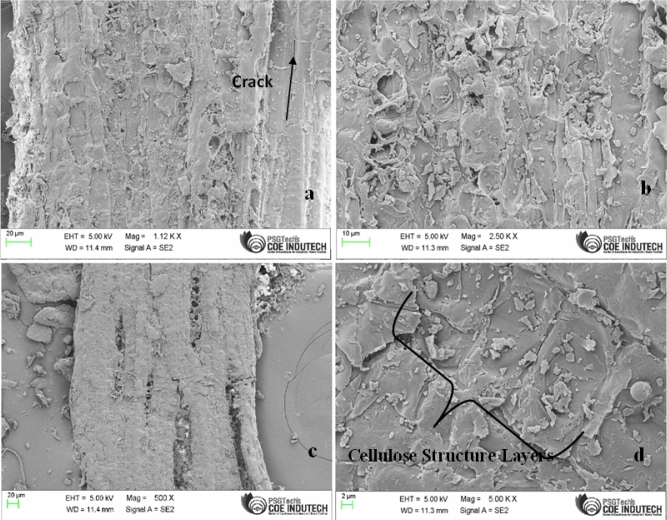
SEM image of Bitter Albizia fiber.

High-precision SEM analysis of natural fiber’s surface morphology revealed microscopic fissures on the BA fiber's surface, roughly halfway along the fiber’s length (Fig. 2a)^14^. The exterior of the BA fiber surface was showed rough (Fig. 2b) in nature. This can increase the area of contact with resin during composite preparation which increase the mechanical properteis of the composites. The presence of white substance in the BA fiber zone (Fig. 2c) shows the existence of amorphous cellulose, lignin and hemicellulose, content^28^. The cellulode sturcture layer are tighly packed. This increase the stiffness of fiber. The higher stiffness of the fiber might make it easier to better adhere to the fiber-matrix by physical contact between them. The gummy polysaccharides and impurities are presented in between the openings of filaments in the fiber surfance. The scanning images demonstrated the avaliblility of sticky polysaccharides in addition to other contaminants between the spaces occupied by the filaments. (Fig. 2d) that are filtered when the fiber is chemically treated and a strong fiber structure formed thereby enhances the BA fiber and matrix inerfacial adhesion during making of the polymer composites. The BPF diameter was taken using FESEM for various phase lengths varying from 17.5 to 68 μm.

### BAFs chemical analysis

Table 2 lists the chemical characteristics of BAFs and numerous other plant fibers. Natural fibers functional properties are largely determined by their cellular and lignin content value. Plant natural fibers with a higher lignocellulose concentration have a high traction strength and tensile modulus, whereas the fiber with a more lignin content have long life^11^. The precentage volume of cellulose (74.89 wt. percent) present in BAFs has relatively high compared to that found in compositions such as Kenaf of 53.14 wt. percent, Palm oil of 65 wt. percent, coir of 37 wt. percent, Jute of 72 wt. percent^20,28^ and hemp fibers of 74 wt^18^ percent. The presence of higher cellulose content is increased the BA fiber tensile properties. The present of lignin content in BAFs was calculatedd to be 12.80 wt. percent, which is greater than sida rhombifolia fiber of 7.48 wt percent^7^, *Hibiscus vitifolius Plant* Stalk of 8.18 wt.percent^14^ hemp fiber of 4 wt. percent^18^ and flax fiber of 3 wt. percent^29^. The lignin content shows medium in BA fiber This can increased the single fiber tensile strength. The increased presence of wax has a negative impact on fiber/matrix interfacial bonding in all natural fiber. BAFs have a medium wax content of 0.31 wt. percent compared to other natural fibers such as Agave gigantea (0.26 wt. percent)^8^, flax fibers (1.7 wt. percent)^29^ and Jute (0.51 wt. percent)^20^. The BA fiber showed less wax content. This will increased the better bonding between fiber-matrix.

**Table 2:** Comparison of chemical compositions of raw BAFs with various natural fibers.

Fibers name	Cellulose (%)	Hemi-cellulose (%)	Lignin (%)	Wax (%)	Moisture content (%)	Ash (%)	Diameter of fiber (μm)	Refs.
Bitter Albizia	74.89	14.5	12.8	0.31	11.71	19.29	17.5–68	–
Artisdita hystrix	59.54	11.35	8.42	–	–	–	1000–3000	^4^
Sida rhombifolia	75.09	15.43	7.48	0.49	12.02	4.07	–	^7^
Acacia leucophloea	68.09	13.6	17.73	0.55	8.83	0.08	1000–3000	^10^
Sansevieria ehrenbergi	80	11.25	7.80	0.45	10.55	0.6	10–250	^11^
Prosopis juliflora	61.65	16.14	17.11	0.61	9.48	5.2	20	^12^
Furcraea Foetida	68.35	11.46	12.32	0.24	5.43	6.53	200–300	^15^
Hibiscus vitifolius	75.09	13.34	10.42	0.17	11.31	0.94	20.5–86	^14^
Cyperus pangorei	68.5	–	17.88	0.17	9.19	–	133.1 ± 17.5	^16^
Acacia planifrons	73.1	9.41	12.04	0.57	8.21	4.06	–	^21^
Perotis indica	68.4	15.7	8.35	0.32	9.54	4.32	–	^30^
Acacia arabica	68.1	9.36	16.86	0.49	–	–	432–1068	^31^
*Grewia tilifolia*	67.19	17	15	–	2.30	-	–	^32^

### FTIR spectrum

**Figure 3:**
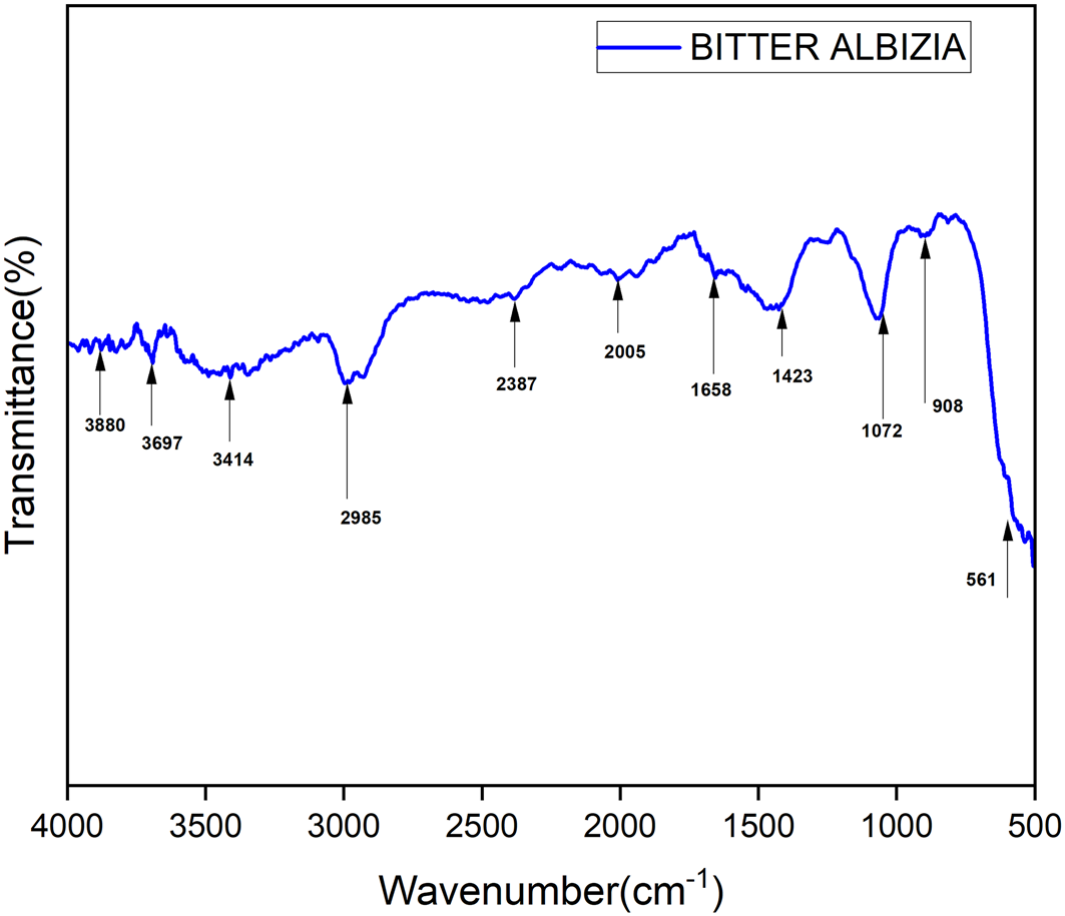
BAFs FTIR spectrum.

FTIR spectrum is used to identify the main active groups existing in BAFs over the wavelength (The specrum range between 500 to 4000 cm^−1^) as showed in Fig. 3. Showing a sharp peak of waveform at 3697 cm^−1^ depicts the free hydroxyl groups with OH expansion, and cellulose hydrogen is represented by the minimum exposure at points of 3880 cm^–1^ and 3414 cm^–1^^11,33^. The expanding C-H bond was shown by a narrow band of 2985 cm^–1^^11,34^. A band visible at 2387 cm^–1^ could be mostly due to lignin ion bonds^11,35^. The tiny peak of 2005 cm^–1^ reflects the presence of CH_2_^33^. It showed the presence of higher crystalline cellulose. In lignin (guaicycl) cellulose, a sharp height of 1658 cm^–1^ correlates to the respiratory guaiacyl ring with a significant C–O stretch^36^. The sharp peak values was very low. This shows the less lignin content in the BA fiber. C–O–C in the from of cellulose is shown by a strong peak at 1072 cm^−1^ and a peak value of 908 cm^−1^ showed β links of BA fiber cellulose^11,37^. OH group bending outside the plane is absorped as high peakc at 561 cm^−1^^35^.

### XRD spectrum

**Figure 4:**
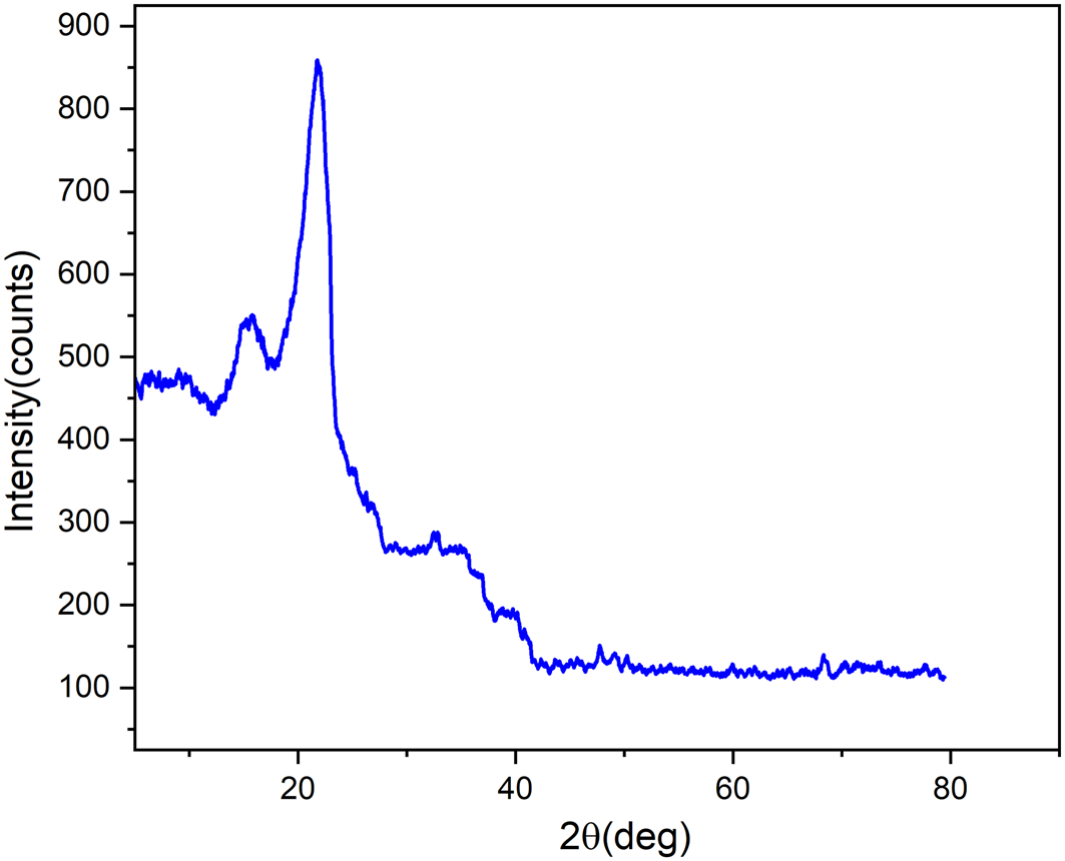
XRT of BAFs.

Figure 4 presence the XRD pattern of BAFs. The Two peaks with angles (2θ = 15.81° and 21.72°) were detected in the diffractogram structure, generally indicating the existence of monoclinic Cellulose-I crystals^10,11^. In all natural plant fibers, crystalline cellulose helps higher tensile stress and also the stability of fiber structure, and this is usually indicated by the CI (crystallinity index)^38^. The BAFs, CI value was found to be 57.20% when tested using the Segal equation mentioned in the scale (Eq. 5) and this number is relatively less compared to that of fibers such as Acacia leucophloea (42%)^10^, Prosopis juliflora (46%)^12^. Furcraea Foetida (42.7%)^28^, Ferula communis (48%)^39^ and Borrassus (38.4%)^40^ fibers. The Scherrer formula^10,11^ was used to calculate the crystallite size BAFs, which was found to be 1.68 nm. Table 3 shows the CI and CS values of BAFs and other fibers in Fig. 4. The BA fiber has higher CI and CS values. This leads to more fiber stiffness.

**Table 3:** Physico-mechanical properties of BAFs and other natural fibers.

Fibers name	Crystalline index (%)	Crystal size (nm)	Tensile strength (MPa)	Tensile modulus (GPa)	Elongation (%)	Density (kg/m^3^)	Refs.
Bitter Albizia	57.20	10.68	513–1226	6.9–9.6	0.8–1.37	1285	**–**
Sida rhombifolia	56.6	2.75	673 ± 14	–	–	1320	^7^
Furcraea foetida	52.6	28.36	623.52 ± 45	6.52 ± 1.9	10.32 ± 1.6	778	^15^
Cyperus pangorei	41	–	196 ± 56	11.6 ± 2.6	1.69	1102	^16^
Passiflora Foetida	67.36	4.23	248–942	11–48	1.38–4.67	1328	^41^
Carica papaya	56.34	15.4	530 ± 11.2	–	1.62 ± 0.02	943	^17^
Perotis Indica	48.3	15	317–1608	8.41–69.61	1.38–4.24	785	^30^
Dichrostachys cinerea	57.82	–	873 ± 14	–	–	1240	^42^
Sida Cordifolia	56.92	18	703.95 ± 23.73	42.84 ± 2.1	2.89 ± 0.24	1330	^43^

### TGA and DTG analysis

**Figure 5:**
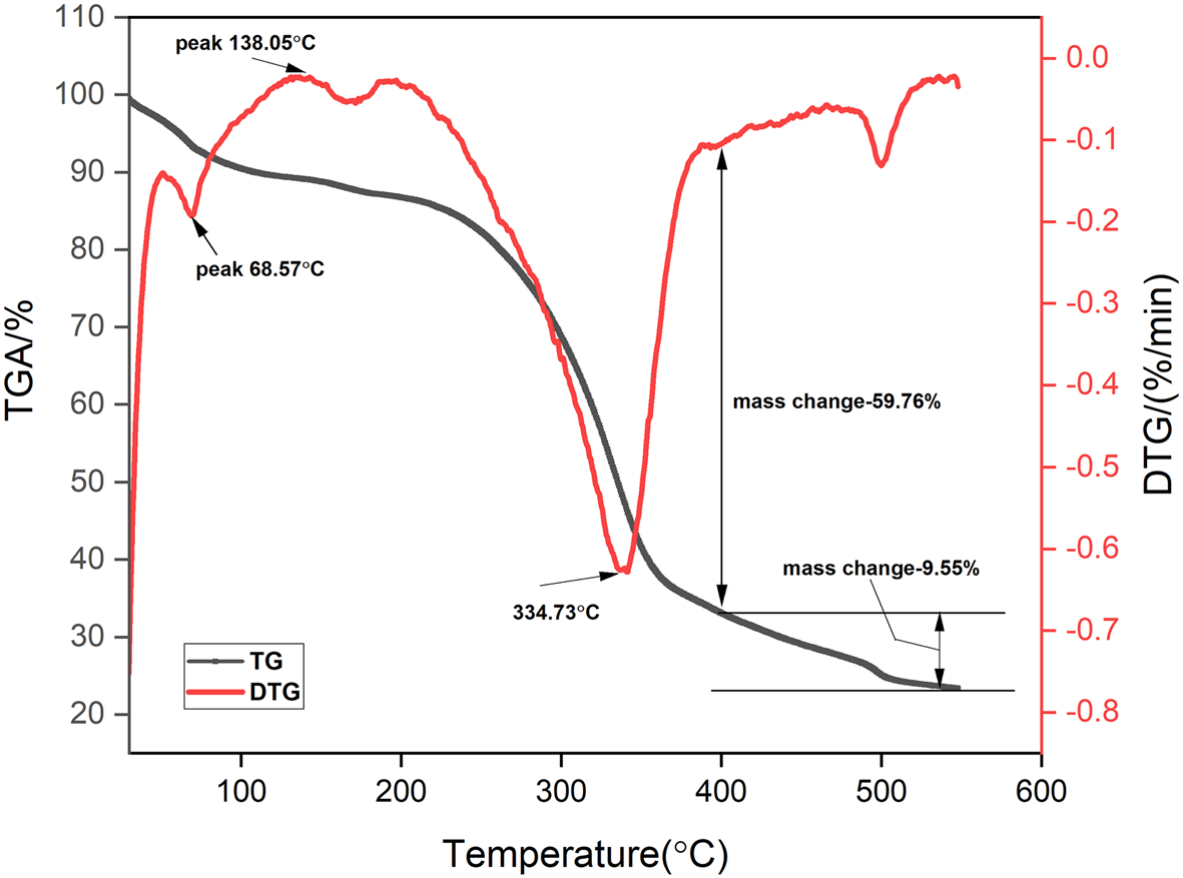
TGA and DTG curves of BAFs.

**Figure 6:**
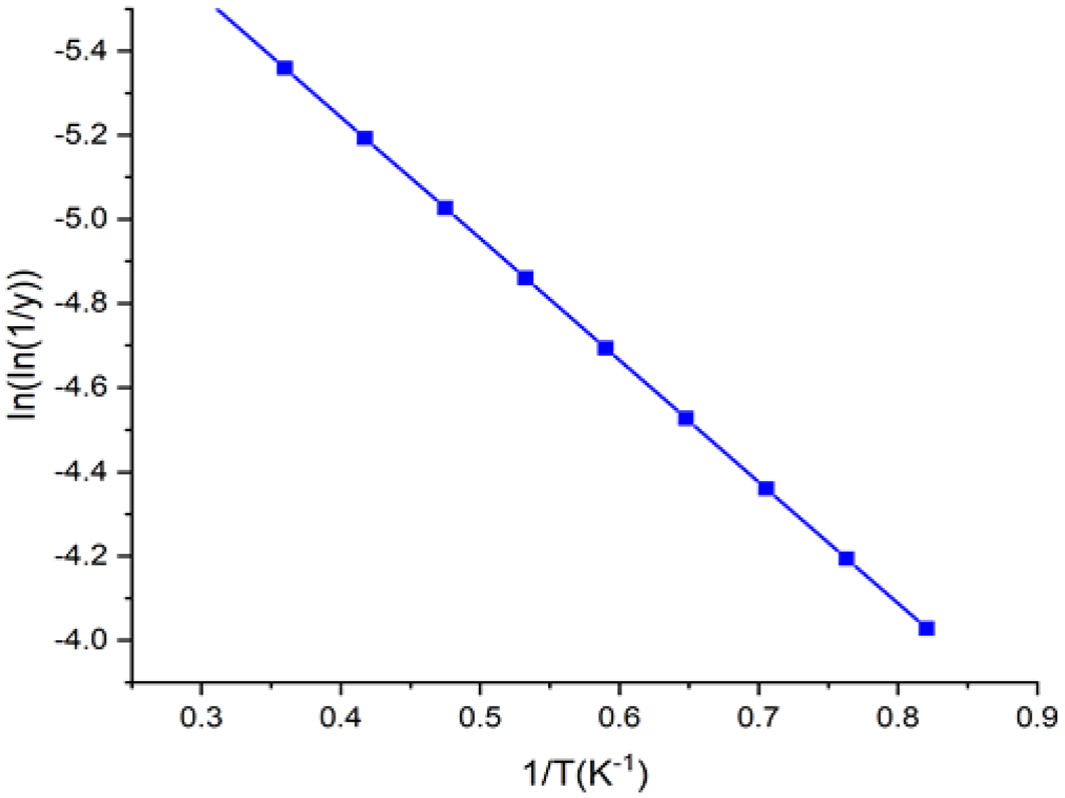
Broido’s plot of BAFs.

The TGA and DTG curves were used to analyze the thermal stability and behavior of BAFs (Fig. 5). The early thermal disintegration fiber samples occurred at 68.57 °C, owing to fibers moisture loss due to evaporation^10,38^. Second phase was occurred from 100 °C to 150 °C with a high temperature of 138.05 °C. Due to a drop in cellulose content, a sudden increase of temperature to 334.73 °C resulted in a large weight loss of roughly 59.76%. Similar peaks at 331.1 °C, 350 °C 328.2 °C, and 346.8 °C have been observed with Sida rhombifolia^7^, Acacia leucophloea^10^ Prosopis juliflora^12^ and Shwetark fibers^36^. Fragrant lignin’s molecular structure makes it more steady in thermally than other natural fiber components^11,16,44^. The residual mass percentage of the fiber at 548 °C was found to be 23.35%. The broido equation (Eq. 6) is used to calculate the activation energy that is associated with thermal degradation of BAFs at temperatures ranging from 80 °C to 360 °C (4). From the structure ln(1/y) compared to T^–1^ shown in Fig. 6, total BAFs regenerative power was showed to be 55.295 kJ/mol. The activation energy is relating to bonding of chemical composition in the natural fiber. The higher value shows higher bonding which could increase the thermal stability like shifting the degradation temperature of composition towards higher level in the TGA curve. The BA fiber have moderate values which shows better thermal stability.

In the DTA curve of TDF given in Fig. 4b four endothermic peaks at 242.03 °C, 364.86 °C, 522.48 °C and 663.77 °C were observed. The first major endotherm ranging between 171.03 °C and 364.86 °C corresponds to thermal depolymerization of hemicellulose and the second major endotherm existing within 333.62 °C and 476.33 °C attributed to the thermal degradation of cellulose. The minor endotherm which ranges between 476.33 °C and 616.20 °C corresponds to the thermal degradation of lignin and the peak at 663.77 °C indicates the temperature at which thermal degradation of wax and other impurities occurs.

The range of temperature over which the thermal decomposition of each chemical constituent occurs is analyzed quantitatively through differential thermal analysis (DTA).

### DTA analysis

**Figure 7:**
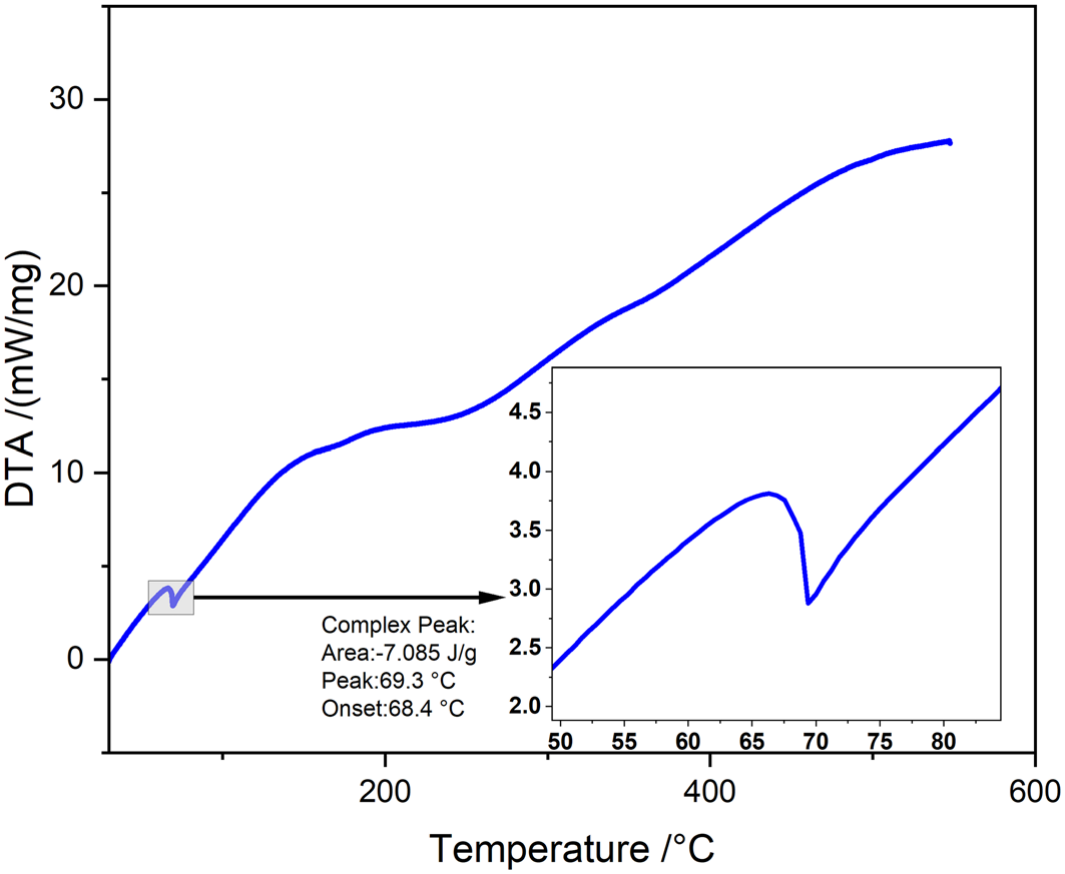
Derivative thermogravimetry analysis of BAFs.

Figure 7 shows the DTA curve of BAFs. The thermal decomposition of chemical composition in BA fiber is investigated quantitatively through DTA as shown in Fig. 7. The DTA with temperature in (°C) shows the complex peak arises at 69.3 °C and the complex onset arises at 68. 4 °C. The area is shown as 7.085 J/g^45^. The two endothermic peaks at 69.3 °C and 68. 4 °C were observed. The first peak at 68. 4 °C showed thermal depolymerization of hemicellulose and lignin, the second peak at 69.3 °C showed thermal degradation of cellulose.

### Mechanical properties of BAF’s

**Figure 8:**
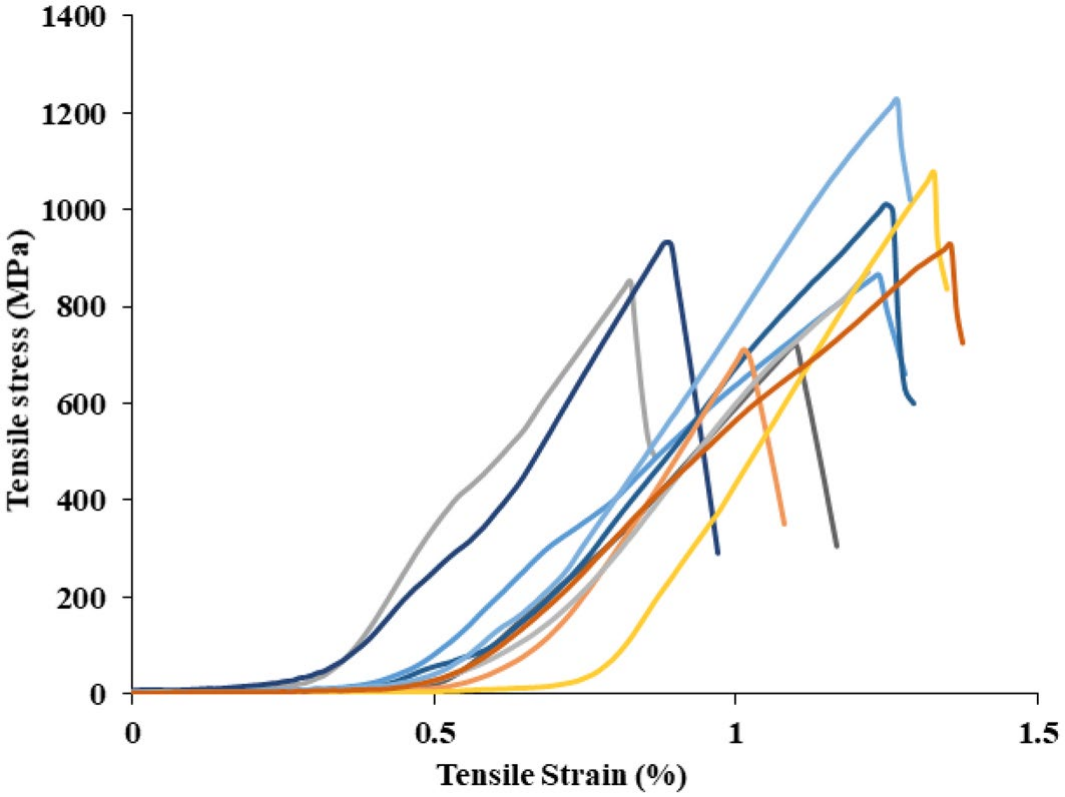
The stress and strain relations for five of BAFs.

Figure 8 presence the BA fiber tensile stress versus strain plots. The tensile propertes of the BAFs was calculated by indirectly from the tensile stress values which was found between 513 and 1226 MPa. The strenght of BAFs was higher than some natual fibers such as palm of 377 MPa, petiole bark of 185.52 MPa, kenaf bast of 427–519, bamboo of 503 and banana of 600 MPa^21,34^. The strain to failure of BAFs was estimated to be 0.8–1.37%, the Youngs modulus of BFAs was estimated from the slope of the tensile stress plots which is 6.9–9.6 GPA. Therefore, the Table 3 shows that it can be concluded that BAFs have a natural fiber that is rather strong. The existence of a high cellulose content in BAFs is responsible for their great potency. The cellulose microfibrils are lodged in the lignin matrix at an angle, known as the microfibrillar angle (α), is another key factor that determines the mechanical characteristics of natural plant fibers^25^. Low microfibrillar angle in natural fiber shows high tensile strength and durability as well as fibers with a higher micro fibrillar angle indicate improved fiber ductility^17^. The BAF has hgiher microfibrillar angle of 11.32° which influences the hgiher mechanical stregnth of fiber and fiber-matrix composites. Plant fiber density, in addition to mechanical qualities, has an essential function in lowering weight and the pace at which it is used in green composites. The physical density of BAFs was discovered as 1285 kg/m^3^. This showed lower value compared to other natural fiber. This BA fiber can used to make light weight composites.

## Conclusion

Physical, mechanical, thermal, morphological, and chemical tests were conducted on biocellulosic fibers from the Bitter Albizia tree for use in composite. The results of these testing was confirmed BAF's ability to strengthen the polymer matrix. The presence of more cellulose content (74.89 wt.%), moisture content (11.71 wt.%), and low wax content of 0.31 wt.% enhances BAF strength and enhances the fiber matrix interfacial bonding. The values of microfibrillar angle (α) of BAFs was showed as 11.32° (α) and plant fibers with α greater than 10° tend to show higher ductility with increasing load. In comparison to most natural fibers, BAFs has a high crystallinity index (57.20%) and a small crystallite size (1.68 nm). This will ideal for the fabrication of polymer composites and increaed the mechanical proeprties. Thermal stability (up to 360 °C) and kinetic activation energy (55.295 kJ / mol) were discovered in BAF by TGA, which are important requirements for compounding. This showed that the BFAs has higher thermal stablity. With this research, it is concluded that a sustainable polymer matrix compoistes can be developed with BAFss that will be useds polymer composite materials in different applications such as car parts, wall panels, sports equipment and building partition boards.

## Data Availability

The datasets and details of plantation and harvesting the all type of Albizia trees are available in Tamil Nadu Agricultural University (https://tnau.ac.in/kvks) Coimbatore, Tamilnadu, India. The report was published by Institute of Forest Genetics and Tree Breeding, Coimbatore. Dr. T. P. Sathishkumar, Associate professor, Department of Mechanical Engineering, Kongu Engineering College, Erode, Tamilnadu, India is collected trees from agricultural land for present research as per the report and guidance.
